# Asialoglycoprotein receptor-targeted perfluorooctylbromide as a targeted contrast agent for evaluating the severity of carbon tetrachloride-induced acute liver damage in rats

**DOI:** 10.3389/fchem.2025.1475026

**Published:** 2025-04-04

**Authors:** Jinhong Yu, Chaofeng Yang, Pengwei Zhang, Min Wei, Yang Li

**Affiliations:** ^1^ Sichuan Key Laboratory of Medical Imaging, Department of Ultrasound, The Affiliated Hospital of North Sichuan Medical College, Nanchong, China; ^2^ Sichuan Key Laboratory of Medical Imaging, Department of Radiology, The Affiliated Hospital of North Sichuan Medical College, Nanchong, China

**Keywords:** Asialoglycoprotein receptor, perfluorooctylbromide, liver damage, ultrasound contrast agent, rats

## Abstract

Asialoglycoprotein receptor (ASGPR) is an endocytic C-type lectin receptor in hepatocytes. Acute and chronic liver diseases can result in the decreased expression and content of this receptor. The objective of this study was to determine whether ASGPR-targeted perfluorooctylbromide (PFOB) can enhance ultrasound imaging signals and evaluate the severity of carbon tetrachloride (CCl4)-induced acute liver damage in rats. The specificity of ASGPR-targeted PFOB for hepatocytes L-02 was investigated *in vitro*. *In vivo*, all rats were treated with either ASGPR-targeted PFOB or PFOB, and ultrasound imaging of the livers was performed to evaluate the effect of these treatments on the imaging signal. The effects of CCl4 injection were also examined by measuring the percentage of apoptotic hepatocytes and ASGPR content. We first confirmed that ASGPR-targeted PFOB can be targeted specifically to hepatocytes L-02. In the healthy rat group, ASGPR-targeted PFOB increased the echo intensity (EI) of the liver by 87.47 dB, which was significantly higher than the EI increase observed with PFOB treatment (37.38 dB; P < 0.001), and the mean elimination times of the contrast agents were 282 ± 13.17 min and 225 ± 10.80 min for the ASGPR-targeted PFOB and PFOB groups, respectively (P < 0.001). In the CCl4-induced acute liver injury group, significant differences were observed in each group before and after administration of ASGPR-targeted PFOB. Significant differences were also observed between the different groups. The degree of reduction in peak EI correlated with the total dose of the CCl4. A decline in ASGPR content was correlated with the severity of acute liver damage using the CCl4-induced model. These findings suggest that ASGPR-targeted PFOB enhances ultrasound imaging and is a reliable tool for assessing the severity of acute liver damage in rats.

## 1 Introduction

Imaging is an important tool in disease research and clinical trials. Molecular ultrasound is an imaging strategy that combines advanced ultrasound technology with targeted contrast agents to evaluate biological processes (Baier et al., 2020; Turco et al., 2017). Molecular ultrasound facilitates both semiquantitative and quantitative assessments of target expression (Baier et al., 2020; Di Paola et al., 2017). Molecular ultrasound contrast agents target specific biomarkers by binding ligands to the surface of the contrast agents. These targeted agents, administered via intravenous injection, accumulate at intended sites that are overexpressing specific biomarkers and enhance the backscattering signal intensity of an ultrasound pulse (Baier et al., 2020).

Ultrasound contrast agents are categorized into microbubble-based and non-microbubble-based agents. Perfluorocarbons (PFCs) are one of the most useful types of non-microbubble-based contrast agents (Lanza et al., 1996; Diaz-Lopez et al., 2010; Li et al., 2013; Kakaei et al., 2023; Holman et al., 2021; Li et al., 2016; Behan et al., 1993). PFCs are 200–400 nm liquid–liquid emulsions consisting of a PFC core encapsulated by a phospholipid monolayer. Compared with the gas core of microbubbles, the liquid composition renders PFCs resistant to mechanical stress and pressure.

In 1977, the efficacy and safety of perfluorooctylbromide (PFOB) as a diagnostic contrast agent for gastroenterography in laboratory animals were first reported by Liu and Long (1977). PFOB is a brominated fluorocarbon and a liquid PFC. Inertness, high oxygen solubility, and stability are characteristics that make it interesting for use in clinic (Diaz-Lopez et al., 2010; Behan et al., 1993). When emulsified with egg-yolk phospholipids, PFOB is stable and suitable for intravenous injection. Unlike water-soluble contrast agents, PFOB does not diffuse into the interstitial space and is not filtered by the kidneys. PFOB is used as a multimodality imaging agent (Li et al., 2013; Li et al., 2016; Behan et al., 1993; Liu and Long, 1977; Barnett et al., 2011; Kuai et al., 2022).

An asialoglycoprotein receptor (ASGPR) is an endocytic C-type lectin receptor in hepatocytes, also known as the galactose or N-acetylglucosamine receptor (Zhuang et al., 2020; Villalta et al., 2015). ASGPR is a liver-specific receptor responsible for removing asialoglycoproteins. Asialoglycoproteins are endogenous glycoproteins in which the sialic acid has been removed by sialidase enzyme activity. The removal of sialic acid makes terminal galactose residues a determining factor in ASGPR recognition [16]. Under normal conditions, ASGPR is mainly expressed on the sinusoidal surface of hepatocytes adjacent to the extracellular space of Disse (Sharma et al., 2021; Zhang et al., 2019). Although the main physiological function of the ASGPR is thought to be clearance of the circulation of glycoproteins containing terminal galactose or N-acetylglucosamine residues, many other physiologic roles, such as removal of apoptotic cells, fibronectin, and immunoglobulin A, have also been reported (McVicker et al., 2002; Rotundo et al., 1999; Lombana et al., 2019). ASGPR also serves as a site of entry for hepatotropic viruses (Zhang et al., 2011).

Arabinogalactan is a ligand of the ASGPR that targets contrast agents only for hepatocytes (Leveille-Webster et al., 1996). We have also reported that this ligand-targeted superparamagnetic PFOB nanoparticle (M-PFOBNP) can improve the R2∗ value of the rat liver parenchyma (Li et al., 2021). In the present study, we prepared galactosylated poly-L-lysine (GalPLL)/PFOB and explored whether this contrast agent could enhance the ultrasound imaging signal and evaluate the degree of damage to the liver according to changes in liver pathology after different doses of carbon tetrachloride (CCl_4_) injection.

## 2 Materials and methods

### 2.1 Preparation of ASGPR-targeted PFOB emulsion

GalPLL was synthesized by reductive amination following our previous description (Li et al., 2021) using a surfactant commixture (Avanti Polar Lipids Inc., AL, United States) containing 90 mol% 1,2-dipalmitoyl-sn-glycero-3-phosphocholine and 10 mol% 1,2-distearoyl-sn-glycero-3-phosphoethanolamine-N-[amino (polyethylene glycol)2000]. The desired amount of surfactant commixture was dissolved in chloroform and evaporated under reduced pressure, forming a dry lipid film under vacuum. The dry lipid film was hydrated by adding 5 mL GalPLL solution. The liposome suspension was combined with liquid PFOB (30% v/v) (Elf Atochem, Paris, France) and emulsified for 4 min using an XL2020 sonicator (Heat Systems Inc, NJ, United States). The PFOB emulsion was prepared through the same process, except 5 mL phosphate-buffered saline (PBS) was added instead of 5 mL GalPLL solution. The mean diameter of the ASGPR-targeted PFOB emulsion was obtained by dynamic light scattering (Malvern Instruments, Malvern, Worcestershire, United Kingdom).

### 2.2 Cells and cell culture

Human hepatocytes L-02 purchased from Harry Bioengineering Co., Ltd (Sichuan, China) were incubated with Roswell Park Memorial Institute (RPMI)-1640 medium (Gibco, Manchester, United Kingdom) containing 10% fetal bovine serum (FBS) at 37°C in a humid incubator with 5% CO_2_.

### 2.3 Cytotoxicity of ASGPR-targeted PFOB

Cell viability was determined by the MTT assay. L-02 cells were seeded into 96-well plates at 2 × 10^3^ per well for 24 h before incubation for 3 h with different volume fractions (1%, 5%, 10%, and 15%) of ASGPR-targeted PFOB solution that were diluted by adding RPMI-1640 medium containing 10% FBS. Cells in the culture medium without ASGPR-targeted PFOB were used as a control. Cell viability was estimated by the addition of 20 μL MTT solution (Sigma, St. Louis, MO, United States) to each well for 4 h. Next, the formazan crystals formed were dissolved by adding 150 μL dimethyl sulfoxide (Sigma, St. Louis, MO, United States). To assess cell viability, optical density (OD) was calculated at 490 nm using an enzyme-linked immunosorbent assay plate reader.

### 2.4 Target assay

L-02 cells (1 × 10^5^ per well) were seeded in a 6-well cell culture plate for 24 h. DiI (5 μL) was added to each well to label cells that were incubated for 15 min. Thereafter, the medium was removed from each well, and the cells were washed five times with PBS. Next, the cells were incubated with fresh medium containing the same volume of fluorescein isothiocyanate (FITC)-labeled ASGPR-targeted PFOB for 0.5 h, 1 h, 1.5 h, and 2 h. Cells in the culture medium with FITC-labeled PFOB were used as a control. The combinations of ASGPR-targeted PFOB and L-02 cells at different time points were observed under an inverted fluorescence microscope (Olympus IX71, Tokyo, Japan).

### 2.5 Animal preparation

Animal experiments were approved by the Ethics Committee of the Affiliated Hospital of North Sichuan Medical College (approval number: K2015118, Nanchong City, Sichuan Province).

#### 2.5.1 Animal model for the enhancement of ultrasound imaging in healthy rats

Twenty male Sprague–Dawley rats (200–250 g, Laboratory Animal Center of North Sichuan Medical College, Nanchong, China) were randomly divided into two groups, with 10 rats in each group. One group was injected with ASGPR-targeted PFOB, whereas the other group was injected with PFOB for control purposes. The two contrast agents (2 mL/kg) were administered into the caudal vein. Anesthesia was induced by intraperitoneal injection of 3% pentobarbital sodium (30 mg/kg).

#### 2.5.2 Animal model of CCl4-induced acute liver damage

Twenty male Sprague–Dawley rats (200–250 g) were randomly divided into control and treatment groups. Acute liver injury was induced in 15 rats by intraperitoneal injection of a mixture of CCl4 and olive oil (1 mL/kg) once a day for 2 days at three concentrations (10%, 30%, and 50%). Five healthy rats were used as control animals and were intraperitoneally injected with an equivalent amount of olive oil. All rats were injected with ASGPR-targeted PFOB via the tail vein at a dose of 2 mL/kg.

### 2.6 *In vivo* ultrasound imaging

All ultrasound images were obtained with a high-resolution ultrasound modality (IU22; Philips Medical Systems, Bothell, WA, United States). Liver images were obtained before and after injections of ASGPR-targeted PFOB and PFOB. The dynamic imaging was obtained at injection and every 10 min to monitor initial changes. Scanning was performed by a sonographer blinded to the contrast administered. All images were acquired with the same parameters (mechanical index [MI], 0.1; depth, 4.0 cm). The echo intensity (EI) of the liver region of interest was measured using an ultrasonic quantitative analysis diagnostic system (“DFY” System, Chongqing Medical University, Chongqing, China) (Ao et al., 2010). The animals were sacrificed by intraperitoneal injection of 3% pentobarbital sodium (100 mg/kg). We verified animal death by respiratory and cardiac arrest lasting more than 1 min.

### 2.7 Histologic evaluation

Liver cell apoptosis was assessed by TUNEL assay. Liver sections of rats in the control and treatment groups were TUNEL-stained using an *in situ* cell death detection kit (Jiangsu KeyGEN BioTECH Co., Ltd, China), complying with the manufacturer’s instructions. At least three different sections were examined for each liver sample, and the numbers of TUNEL-positive cells in 15 sections of five different rats from each group were observed in high-power fields (200-fold magnification). The pathologist was blinded to assess the histology (Yao et al., 2017).

### 2.8 Western blot analysis

Western blotting analysis was performed as previously described with minor modifications (Yao et al., 2017). Briefly, the liver tissues (250 mg) were homogenized, solubilized in ice-cold lysis buffer, and centrifuged (14,000 rpm, 30 min) at 4°C. The homogenate supernatant was collected for subsequent analysis. Protein concentrations were measured using the bicinchoninic acid method. Aliquots of the suspension were resolved on a 10% sodium dodecyl sulfate-polyacrylamide gel, and proteins were transferred to polyvinylidene difluoride membranes. ASGPR rabbit anti-human antibody and rabbit anti-human β-actin antibody (Santa Cruz Biotechnology, Inc, United States) were used as primary antibodies. Goat anti-rabbit IgG (Beyotime Institute of Biotechnology, China) was used as the secondary antibody. Following multiple washes in PBS, the blots were dried and detected by enhanced chemiluminescence. The enhanced chemiluminescence exposed blots were quantified using Quantity One software (Bio-Rad, Hercules, CA).

### 2.9 Statistical analysis

All the data are expressed as mean ± standard deviation. We used independent samples t-test and one-way analysis of variance for variables to compare differences in treatment between the two groups. SPSS software (SPSS Inc., Chicago, IL, United States) was used for statistical analysis. A P value <0.05 was considered statistically significant.

## 3 Results

### 3.1 The diameter of ASGPR-targeted PFOB

**FIGURE 1:**
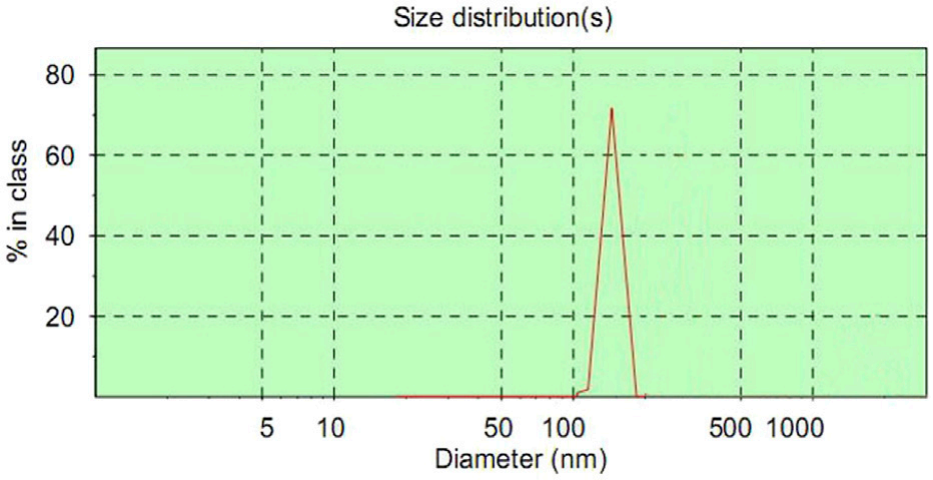
The diameter of ASGPR-targeted PFOB.

The mean diameter of ASGPR-targeted PFOB was 118 ± 22.6 nm, and the diameter distribution was nearly symmetric (Figure 1). Furthermore, the mean diameter of PFOB was 114 ± 35.8 nm, and the diameter distribution was also nearly symmetric. The difference in size between these two contrast agents was not statistically significant (P > 0.05).

### 3.2 Cytotoxicity

**FIGURE 2:**
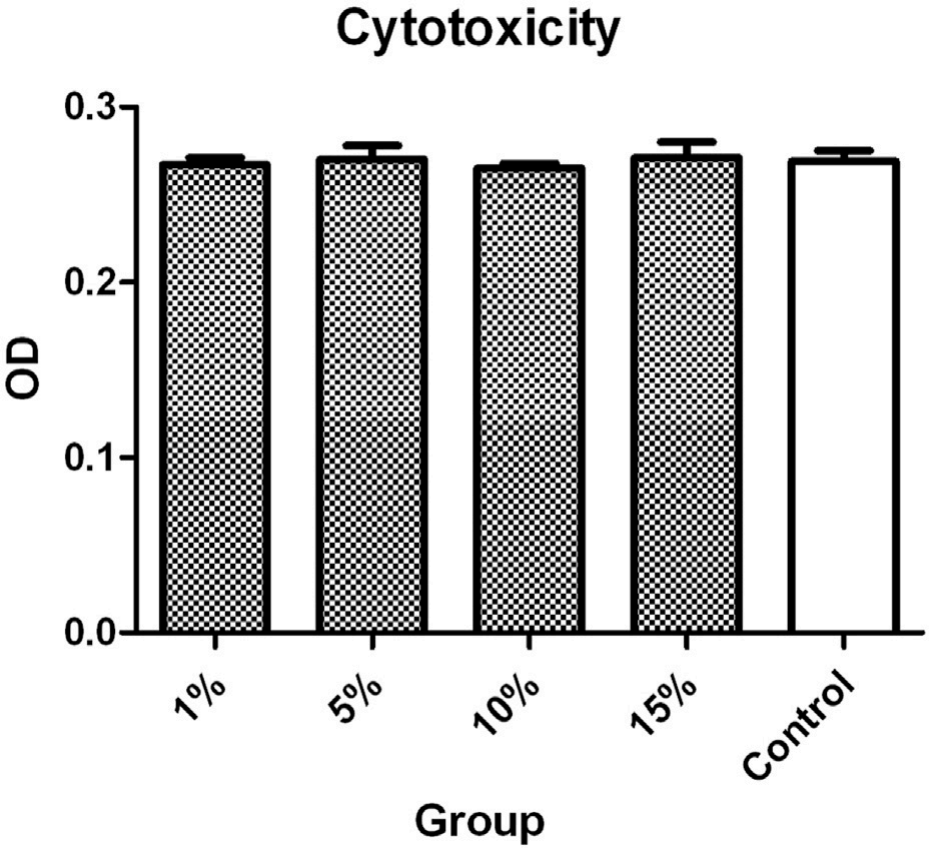
The MTT assay indicated that the difference in the OD of L-02 cells incubated with different concentrations of ASGPR-targeted PFOB solution was not statistically significant.

The ODs of L-02 cells incubated with 1%, 5%, 10%, and 15% v/v ASGPR-targeted PFOB solution were 0.267 ± 0.004, 0.270 ± 0.008, 0.265 ± 0.003, and 0.271 ± 0.009, respectively. The OD of the control group was 0.269 ± 0.006. The MTT assay indicated that the differences in OD of L-02 cells incubated with different concentrations of ASGPR-targeted PFOB solution were not statistically significant (P > 0.05) (Figure 2).

### 3.3 The combination of ASGPR-targeted PFOB and L-02 cells

**FIGURE 3:**
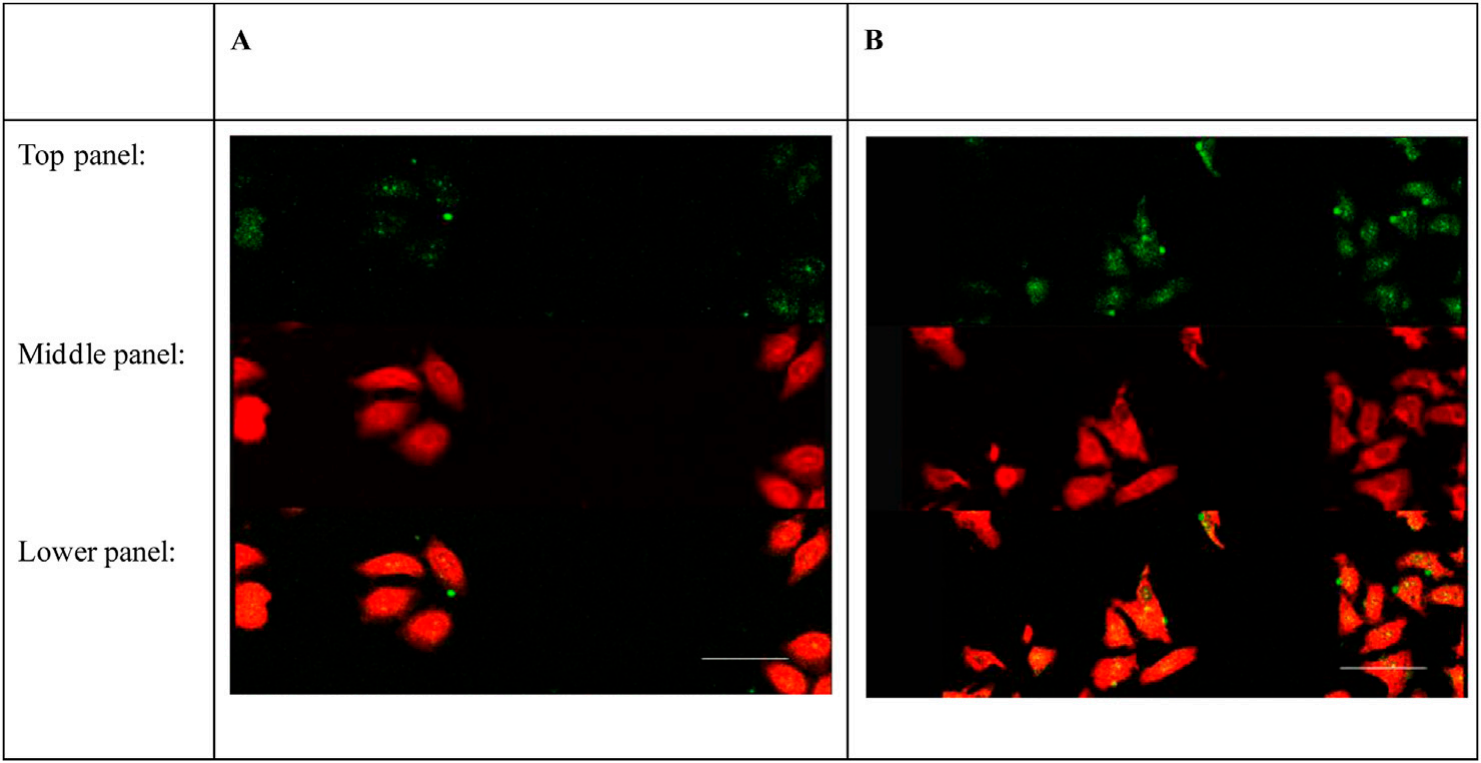
ASGPR-targeted PFOB binds to L-02 cells *in vitro*. The image shows the targeted PFOB on L-02 cells for 0.5 h **(A)** and 1 h **(B)**, respectively. The photomicrographs were taken at 400× magnification. Scale bar: 50 μm. Top panel: L-02 cells after incubation with FITC-labeled ASGPR-targeted PFOB. Middle panel: L-02 cells after staining with DiI. Lower panel: Merged images of **(A, B)**.

A few ASGPR-targeted PFOB conglutinated to the L-02 cells (Figure 3A) 0.5 h after the application of fresh medium containing the same volume as ASGPR-targeted PFOB. However, many ASGPR-targeted PFOB combined with L-02 cells were observed after 1 h (Figure 3B). No visible increase was observed in the conglutination of ASGPR-targeted PFOB to L-02 cells after 1.5 h and 2 h. However, no conglutination was observed between PFOB and L-02 cells.

### 3.4 *In vivo* ultrasound imaging

#### 3.4.1 Ultrasound imaging in healthy rats

**FIGURE 4:**
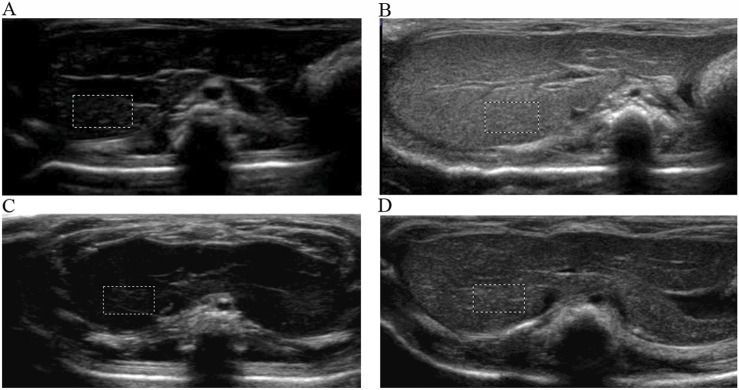
Ultrasound imaging of ASGPR-targeted PFOB and PFOB in the liver. The EI of the liver is homogeneous before the introduction of agents **(A, C)**. Images show that the EI increases in the liver parenchyma for both ASGPR-targeted PFOB **(B)** and PFOB **(D)** 1 h after the administration of agents, whereas it increases dramatically and more homogeneously in the liver of rats treated with ASGPR-targeted PFOB **(B)**.

**FIGURE 5:**
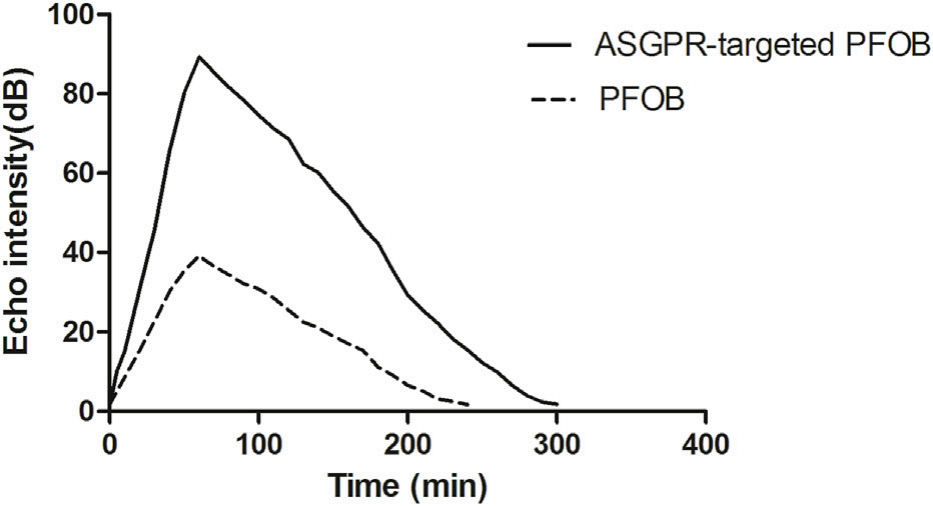
Graph showing the relationship between the EI of liver parenchyma and time after the administration of contrast agents.

ASGPR-targeted PFOB increased the EI of the liver from 1.81 ± 0.10 dB to 89.28 ± 3.34 dB (P < 0.001), resulting in an average increase of 87.47 dB. PFOB increased the EI of the liver from 1.78 ± 0.11 dB to 39.16 ± 2.30 dB (P < 0.001), resulting in an average increase of 37.38 dB (Table 1). A dramatic increase in the EI of the entire hepatic parenchyma was detected on images enhanced with ASGPR-targeted PFOB, and the peak enhancement of the liver in the ASGPR-targeted PFOB group was much greater than that in the PFOB group. Serial measurement of EI revealed that the maximal EI of the liver in rats occurred approximately 1 h after administration of ASGPR-targeted PFOB or PFOB, compared with that in the unenhanced image (Table 1) (Figures 4, 5). Elimination of ASGPR-targeted PFOB in the liver was significantly slower than that of PFOB. The mean elimination times of the contrast agents were 282 ± 13.17 min and 225 ± 10.80 min in the ASGPR-targeted PFOB group and PFOB group, respectively (P < 0.001) (Table 1) (Figure 5).

**TABLE 1:** Enhancement characteristics of rat livers in the two groups.

Variable	ASGPR-targeted PFOB	PFOB	P value
Mean EI of pre-contrast (dB)	1.81 ± 0.10	1.78 ± 0.11	0.518
Maximal EI of post-contrast (dB)	89.28 ± 3.34	39.16 ± 2.30	<0.001
Elimination time of contrast agent (min)	282 ± 13.17	225 ± 10.80	<0.001

#### 3.4.2 Ultrasound imaging of CCl4-induced acute liver damage

**FIGURE 6:**
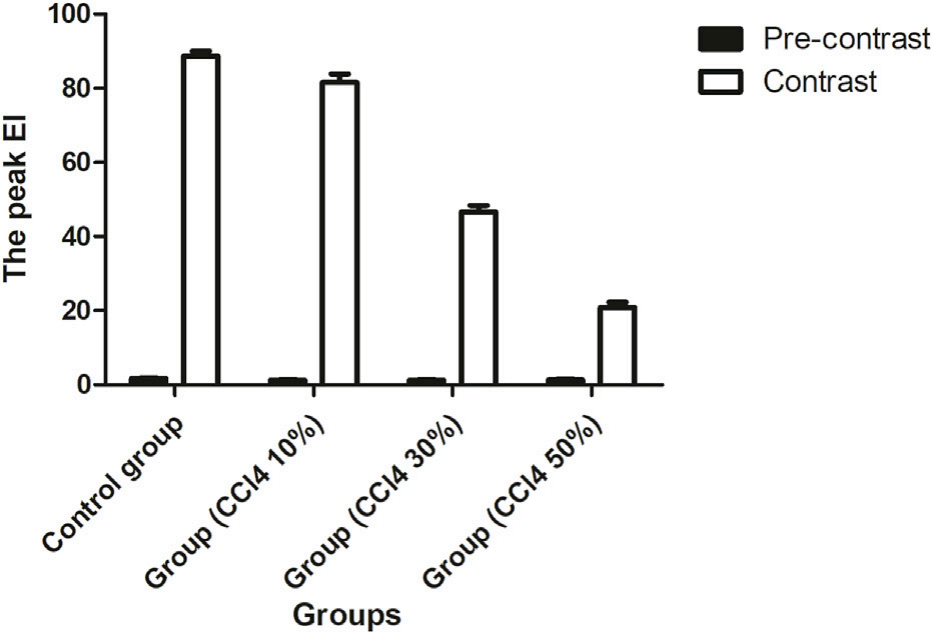
Changes in EI before and 1 h after administration of ASGPR-targeted PFOB in the control and injured rat groups.

In Figure 6, the EIs of the liver were 1.81 ± 0.97 dB, 1.27 ± 0.11 dB, 1.28 ± 0.10 dB, and 1.39 ± 0.12 dB for the control and injured rats with three different concentrations of CCl4, respectively. The EI of the injured liver was lower than that of the control before the administration of ASGPR-targeted PFOB (P < 0.001); however, no obvious statistical difference was observed among the treatment groups (F = 2.201, P = 0.153). The peak EI of the injured liver decreased significantly 1 h after administration of ASGPR-targeted PFOB. The peak EI of the control liver was 88.68 ± 1.36 dB, and those of the injured livers were 81.61 ± 2.30 dB, 46.63 ± 1.77 dB, and 20.84 ± 1.50 dB. Furthermore, different pairwise comparisons revealed significant differences between the different groups (P < 0.001). The degree of reduction in peak EI correlated with the total dose of CCl4.

### 3.5 Injection of CCl4 caused hepatocyte apoptosis

**FIGURE 7:**
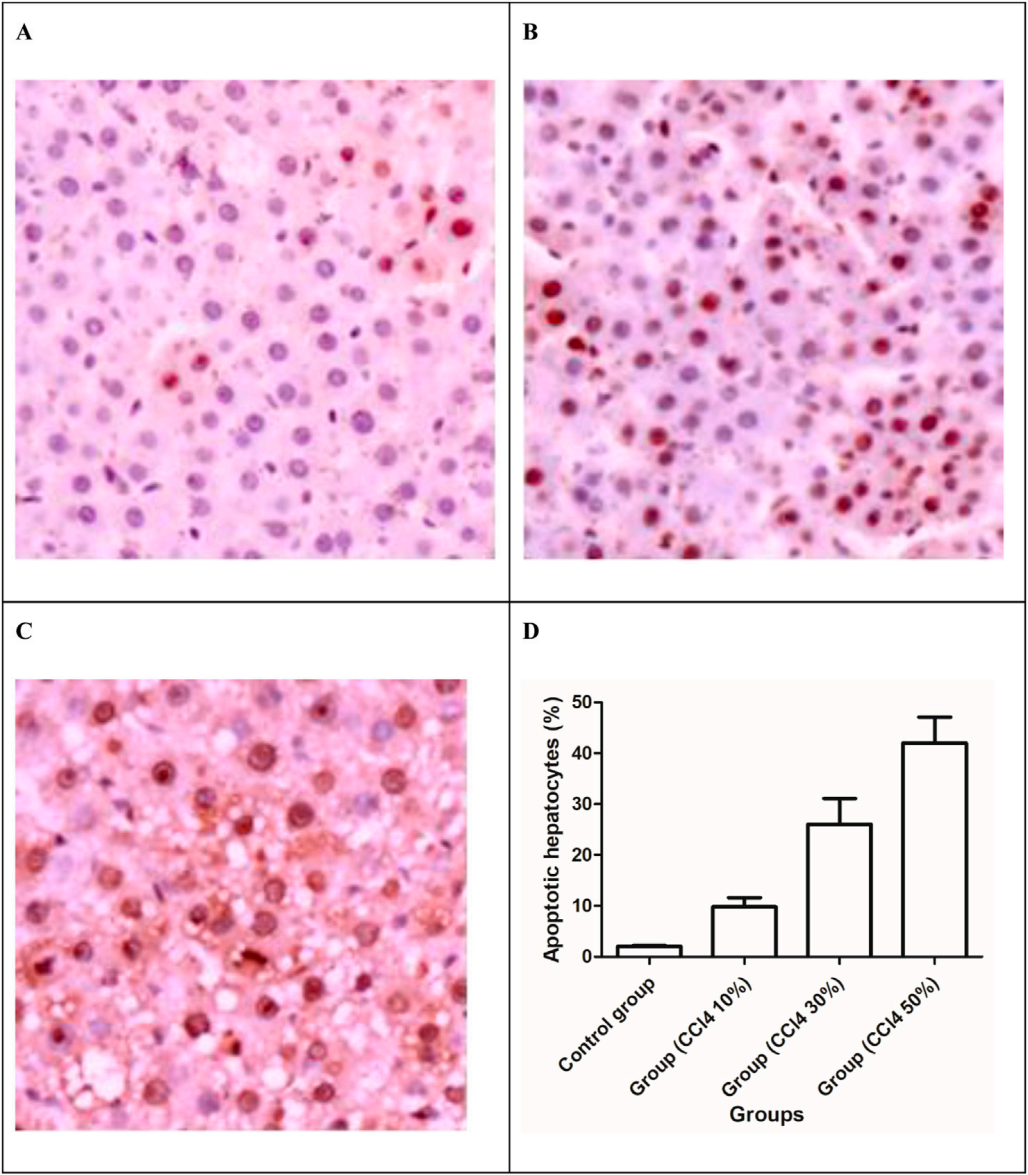
TUNEL apoptosis assay images of liver tissues. Brown dots were deemed apoptosis-positive cells (magnification ×200). **(A)** 10% solution of CCl4, **(B)** 30% solution of CCl4, **(C)** 30% solution of CCl_4_, and **(D)** apoptotic hepatocytes (%) in the liver of the control and injured rat groups.

With the increase in the total drug dosage, the apoptotic hepatocytes induced by CCl4 significantly increased (P < 0.001) and were manifested by the upsurge of TUNEL-positive cells (Figure 7). The liver cell apoptosis induced in rats receiving 50% CCl4 injection was approximately 4.8-fold higher than in rats receiving 10% CCl4 injection.

### 3.6 Measurement of ASGPR protein content

**FIGURE 8:**
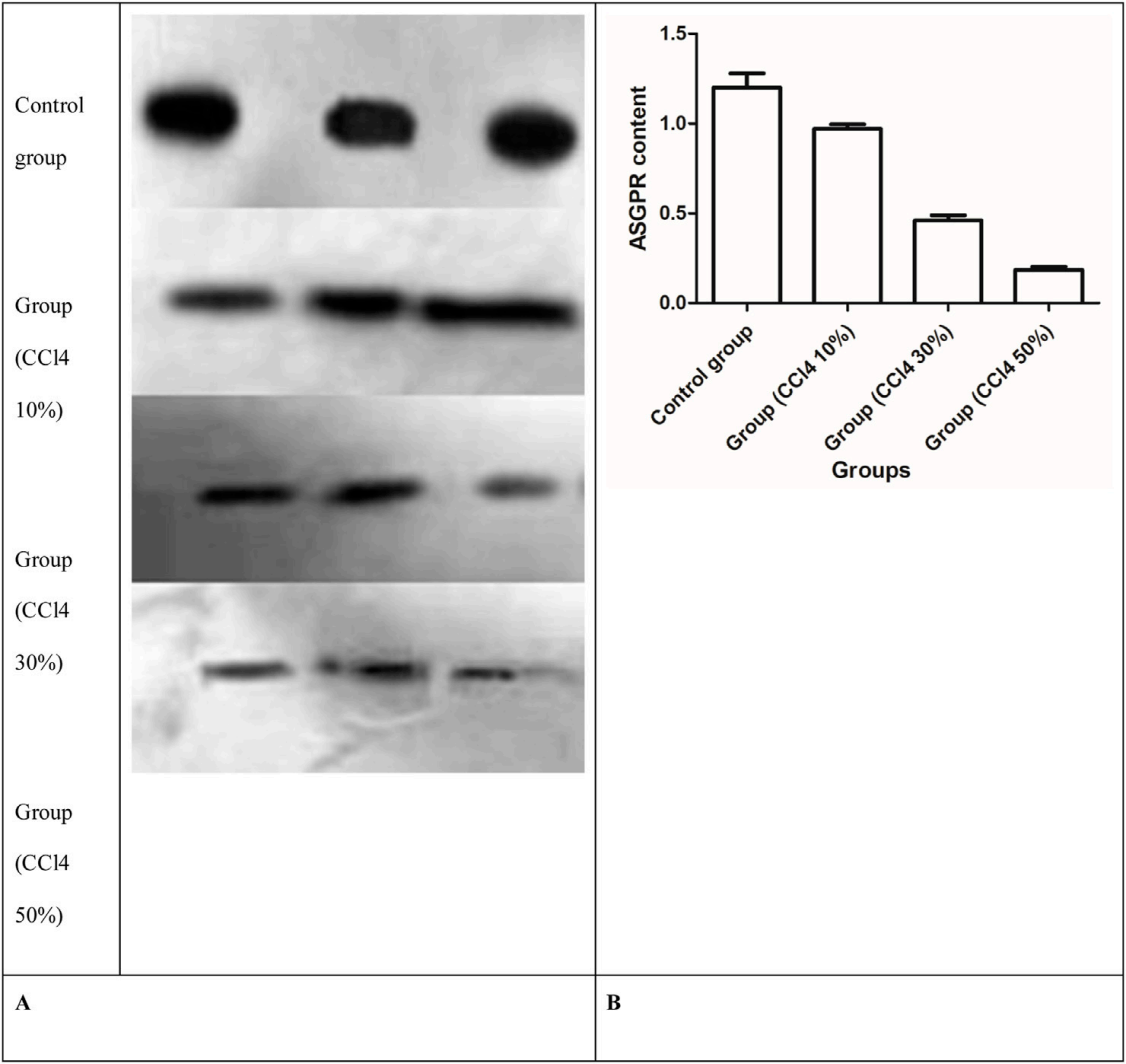
Western blot analysis of ASGPR content in rat liver. **(A)** Representative immunoblots of solubilized liver fractions from control and treatment groups that were loaded onto gels and resolved by SDS-PAGE, followed by transblot analysis. **(B)** ASGPR content in the control and injured rat groups.

After 2 days of injecting three different concentrations of CCl4, quantitative analysis of the immunoblots showed changes in the amount of the 47-kDa subunit of the ASGPR, and the ASGPR content decreased compared with that in the control group (Figure 8). The decrease between the control and injured rat groups was statistically significant. Significant differences were also observed in the ASGPR content among groups (P < 0.001) (Figure 8).

## 4 Discussion

Our study established that ASGPR-targeted PFOB can specifically target L-02 cells. The findings are supported by the *in vitro* target assay and the *in vivo* ultrasound imaging data. ASGPR-targeted PFOB can be used to study ASGPR biology in animals with intact and CCl4-induced acute liver damage. The agent PFOB was conjugated with GalPLL. We also demonstrated that the ASGPR content decreased and that the changes were exacerbated when CCl4-induced pathological features were enhanced.

Current macroscopic imaging systems, which include computed tomography, magnetic resonance (MR) imaging, and ultrasound, can provide anatomical and limited physiological information and are widely used in clinical practice (Giardino et al., 2017; Lanza et al., 2000). In our study, PFOB was combined with GalPLL to obtain a targeted contrast agent for ultrasound imaging. The targeted PFOB considerably increased the EI of the liver 1 h after the administration of targeted PFOB by 87.47 dB. The peak enhancement of the liver in the ASGPR-targeted PFOB group was much greater than that in the PFOB group. Compared with previous results, the peak time was different (Satterfield et al., 1993). Although the exact reason for the different peak times is unclear, the difference in PFC preparation might be a possible reason. PFCs were emulsified in egg-yolk phospholipid and pluronic-F68 in previous studies. However, in our study, PFOB was emulsified in 1,2-dipalmitoyl-sn-glycero-3-phosphocholine and 1,2-distearoyl-sn-glycero-3- phosphoethanolamine-N-[amino (polyethylene glycol)2000].

Previous studies showed that PFOB caused prolonged enhancement of the liver and spleen and increased the conspicuity of liver tumors (Behan et al., 1993). The contrast enhancement could last approximately 3 h. In our study, ASGPR-targeted PFOB increased the EI of the liver. The elimination of ASGPR-targeted PFOB in the liver was significantly slower than that of PFOB. Once the targeted contrast agents accumulate at the site of interest, the pathologic tissue can be enhanced by increased acoustic backscatter; therefore, molecular ultrasound imaging indicates the presence of biomarkers associated with disease. Monoclonal antibodies and fragments, polysaccharides, peptides, drugs, and aptamers can be used as ligands, which may be attached covalently or non-covalently to the contrast agent (Baier et al., 2020). The liquid PFC core is surrounded by a phospholipid monolayer that can be functionalized with various agents, including homing ligands, drugs, and imaging agents. Lanza et al. reported on a ligand-based ultrasound contrast agent that uses fibrin-targeted PFC to target arterial thrombi significantly to improve the signal-to-noise ratio for the acoustic imaging of arterial thrombi in dogs (Lanza et al., 1996). Lanza et al. have also demonstrated that the tissue factor-targeted PFC enhances the detection of carotid artery injury in pigs (Lanza et al., 2000). Owing to the accumulation of PFCs in the tunica media, high echogenicity occurred in the injured part of the artery. Marsh et al. proposed a theoretical model to quantify the relationship between the concentration of PFOB droplets and the contrast enhancement of targeted surfaces (Marsh et al., 2007). The model showed that once the droplets attach to the surface, the reflectivity of the PFOB droplets increases. In our study, PFOB was conjugated with GalPLL. Targeted PFOB was preferred for use as an ultrasound contrast agent, compared with PFOB, and yielded a higher ultrasound imaging signal for rat liver.

More than 100,000 functional ASGPRs are randomly distributed on the basolateral plasma membrane of a normal hepatocyte (Leveille-Webster et al., 1996). The galactose derivative complexes can specifically target hepatocytes through the ASGPR on the cell surface. ASGPR function declines not only during liver regeneration but also in various acute and chronic liver diseases (Zhang et al., 2019; Leveille-Webster et al., 1996). GalPLL has been used to mediate specific gene transfer into hepatocytes. The receptor-mediated targeting of plasmid DNA to hepatocytes has been achieved through a plasmid DNA–GalPLL complex (Nishikawa et al., 1998). In our study, GalPLL was synthesized by reductive amination, binding the phospholipid shell of PFOB as the targetable ligand to hepatocytes. The enhancement mechanism of the targeted PFOB in our study is different from other ultrasound microbubble agents that depend on a low-frequency resonance to enhance ultrasonic backscatter (Lanza et al., 2000). We have found that CCl4-induced hepatotoxicity markedly decreased ASGPR content. Our results also showed that apoptotic hepatocytes significantly increased with the increase in the total dosage of CCl4. This suggests that the impairment of ASGPR function is related to the degree of liver injury. Of note, our research group functionalized M-PFOBNP by targeting GalPLL and transformed it into an ASGPR-targeted MR contrast agent that can be used to study ASGPR biology in intact animals (Li et al., 2021). The molecular imaging of ASGPR-targeted PFOB may provide a tool to evaluate the change in ASGPR function by ultrasound imaging signals and a quantitative *in vivo* measurement of ASGPR function that may be clinically useful for monitoring disease states and therapeutic responses. Furthermore, most malignant tumors lack ASGPRs; therefore, ASGPR-targeted PFOB is expected to provide excellent contrast between normal liver and malignant tumors.

Our study has one limitation. Reporting from our initial experience, the absence and presence of ASGPR-targeted PFOB at four time points, 0.5 h, 1 h, 1.5 h, and 2 h, were not clearly represented owing to the lack of dynamic observation.

In conclusion, this study indicates that ASGPR-targeted PFOB can improve the enhancement of ultrasound imaging signals and may provide information on ASGPR function. Although targeted PFOB is specific for ASGPR imaging, surface modification of these small PFOB droplets can allow the development of various receptor- and antibody-specific agents for disease imaging.

## Data Availability

The original contributions presented in the study are included in the article/supplementary material; further inquiries can be directed to the corresponding author.
